# World Journal of Emergency Surgery reviewer acknowledgement 2015

**DOI:** 10.1186/s13017-016-0068-3

**Published:** 2016-03-11

**Authors:** Fausto Catena, Frederick Moore

**Affiliations:** Department of Emergency and General Surgery, Parma University Hospital, Parma, Italy; Department of Surgery, University of Florida, Gainesville, Florida USA

## Abstract

The editors of *World Journal of Emergency Surgery* would like to thank all our reviewers who have contributed to the journal in volume 10 (2015).

**Fikri Abu-Zidan**

United Arab Emirates

**Ferdinando Agresta**

Italy

**Jamir Arlikar**

India

**Erman Aytac**

Turkey

**Miklosh Bala**

Israel

**Cino Bendinelli**

Australia

**Jasneet Bhullar**

United States of America

**Walter Biffl**

United States of America

**Luigi Bonavina**

Italy

**Clay Cothren Burlew**

United States of America

**Fabio Campanile**

Italy

**Osvaldo Chiara**

Italy

**Mircea Chirica**

France

**Ian Civil**

New Zealand

**Federico Coccolini**

Italy

**Raul Coimbra**

United States of America

**Lidia Dalfino**

Italy

**James Davis**

United States of America

**Marc De Moya**

United States of America

**Nicola de'Angelis**

France

**Marc deMoya**

United States of America

**Salomone Di Saverio**

Italy

**Jose Diaz**

United States of America

**Paul Eustace**

Ireland

**Eriberto Farinella**

Italy

**Paula Ferrada**

United States of America

**Andreas Fette**

Germany

**Gustavo Fraga**

Brazil

**Ralf Herbert Gahr**

Germany

**Sanjay Gupta**

India

**Daithi Heffernan**

United States of America

**Rao Ivatury**

United States of America

**Jeffry Kashuk**

Israel

**Radwan Kassir**

France

**Kenji Kawamukai**

Italy

**Michael Kelly**

Australia

**Peter Kim**

United States of America

**Fernando Kim**

United States of America

**Boris Kirshtein**

Israel

**Yoram Kluger**

Israel

**Rosemary Kozar**

United States of America

**Rifat Latifi**

United States of America

**I. Michael Leitman**

United States of America

**Ari Leppaniemi**

Finland

**Lawrence Lottenberg**

United States of America

**Ronald Maier**

United States of America

**Mark Malangoni**

United States of America

**Ingo Marzi**

Germany

**Oner Mentes**

Turkey

**David Muckart**

South Africa

**Pradeep Navsaria**

South Africa

**Vinesh Padayachy**

South Africa

**Abhijeet Patil**

United Kingdom

**Edoardo Picetti**

Italy

**Stefano Rausei**

Italy

**Joao Rezende-Neto**

Canada

**Sandro Rizoli**

Canada

**Giorgio Rossi**

Italy

**Boris Sakakushev**

Bulgaria

**Massimo Sartelli**

Italy

**Thomas Scalea**

United States of America

**Helmut Segovia Lohse**

Paraguay

**Pablo Serrano**

Canada

**Mohd Sheikh**

United States of America

**Kjetil Soreide**

Norway

**Philip Stahel**

United Kingdom

**Angela Sultana**

Malta

**Peep Talving**

Estonia

**Gia Tomadze**

Georgia

**Gregorio Tugnoli**

Italy

**George Velmahos**

United States of America

**Imtiaz Wani**

India

**Dieter Weber**

Australia

